# A New Plan for Drainage of Wounds

**Published:** 1877-01

**Authors:** 


A New Plan for Drainage of Wounds.—In a paper read before the British Association, Mr. Chiene advocated the substitution of hanks of catgut prepared in carbolic acid solution for the india-rubber tubes in ordinary use. After illustrating, by a reference to the manner in which field drains were laid, that it was not absolutely necessary that a drain-pipe should be either tubular or patent, he expressed his conviction that a hank or skein of catgut would act, by capillary attraction, even more powerfully than an ordinary tubular piece of india-rubber. It would have the additional advantage that it would not need to be shortened or removed, as the granulations of the parts might be trusted to absorb it. Four cases illustrated the paper. One was an amputation at the ankle, drained by three hanks of catgut. In this, putrefaction took place, and the drain was not a success. One, an excision of knee, for anchylosis, healed up well; and two others, in which small tumors had been excised with antiseptic precaution, had done well also. From these somewhat scanty data, the conclusions had been drawn. The size of the skeins was to vary with their number and the depth of wound to be drained, and the most careful antiseptic precautions were to be taken.—*Medical and Surgical Reporter.*

